# Nonnormative Eating Behaviors and Eating Disorders and Their Associations With Weight Loss and Quality of Life During 6 Years Following Obesity Surgery

**DOI:** 10.1001/jamanetworkopen.2022.26244

**Published:** 2022-08-11

**Authors:** Anja Hilbert, Christian Staerk, Annika Strömer, Thomas Mansfeld, Johannes Sander, Florian Seyfried, Stefan Kaiser, Arne Dietrich, Andreas Mayr

**Affiliations:** 1 Integrated Research and Treatment Center AdiposityDiseases, Behavioral Medicine Research Unit, Department of Psychosomatic Medicine and Psychotherapy, University of Leipzig Medical Center, Leipzig, Germany; 2 Department of Medical Biometrics, Informatics and Epidemiology, University Hospital Bonn, Bonn, Germany; 3 Department of General Surgery, Asklepios Clinic, Hamburg, Germany; 4 Schön Klinik Hamburg Eilbek, Obesity Clinic, Hamburg, Germany; 5 Department of General, Visceral, Transplant, Vascular and Pediatric Surgery, University Hospital, University of Würzburg, Würzburg, Germany; 6 Department of Visceral, Pediatric and Vascular Surgery, Hospital Konstanz, Konstanz, Germany; 7 Department of Surgery, Clinic for Visceral, Transplantation, Thoracic and Vascular Surgery, University Hospital Leipzig, Leipzig, Germany

## Abstract

**Importance:**

Individuals with severe obesity presenting for obesity surgery (OS) frequently show nonnormative eating behaviors (NEBs) and eating disorders (EDs), but the long-term course and prospective associations with weight loss and health-related quality of life (HRQOL) remain unclear.

**Objective:**

To examine the prevalence and prospective relevance of presurgical and postsurgical NEBs and EDs according to the *Diagnostic and Statistical Manual of Mental Disorders, Fifth Edition*, diagnosed through clinical interview, for weight loss and HRQOL up to 6 years following OS.

**Design, Setting, and Participants:**

In the prospective, multicenter Psychosocial Registry for Obesity Surgery cohort study, patients seeking OS were recruited at 6 OS centers in Germany and assessed at baseline before surgery and at 6 months and 1 to 6 years after surgery. From a consecutive sample of 1040 volunteers with planned OS from March 1, 2012, to December 31, 2020, a total of 748 (71.92%) were included in this study. Across follow-up, 93 of the 748 patients (12.43%) dropped out. Data were analyzed from April to November 2021.

**Interventions:**

Laparoscopic Roux-en-Y gastric bypass or sleeve gastrectomy.

**Main Outcomes and Measures:**

Both NEBs and EDs were identified using the Eating Disorder Examination interview. Main outcomes were the percentage of total body weight loss (%TBWL) and HRQOL (Impact of Weight on Quality of Life-Lite; range, 0-100, with 0 indicating worst and 100 indicating best).

**Results:**

In 748 patients undergoing OS (mean [SD] age, 46.26 [11.44] years; mean [SD] body mass index [calculated as weight in kilograms divided by height in meters squared], 48.38 [8.09]; 513 [68.58%] female), the mean (SD) %TBWL was 26.70% (9.61%), and the mean (SD) HRQOL improvement was 35.41 (20.63) percentage points across follow-up. Both NEBs and EDs were common before surgery, with postsurgical improvements of varying degrees. Whereas NEBs and EDs did not reveal significant prospective associations with %TBWL, loss-of-control eating at follow-up was concurrently associated with lower %TBWL (estimate, −0.09; 95% CI, −0.14 to −0.04). Loss-of-control eating (estimate, −0.10; 95% CI, −0.17 to −0.03 percentage points) and binge-eating disorder of low frequency and/or limited duration (estimate, −6.51; 95% CI, −12.69 to −0.34 percentage points) at follow-up showed significant prospective associations with lower HRQOL.

**Conclusions and Relevance:**

This cohort study found prospective relevance of loss-of-control eating and binge-eating disorder of low frequency and/or limited duration for reduced long-term HRQOL following OS. These findings underline the importance of monitoring both NEBs, especially loss-of-control eating, and EDs in the long term postsurgically to identify patients in need of targeted prevention or psychotherapy.

## Key Points

**Question:**

Are eating disorders and nonnormative eating behaviors prospectively associated with weight loss and health-related quality of life (HRQOL) up to 6 years following obesity surgery?

**Findings:**

In a multicenter cohort study of 748 patients who presented for obesity surgery, eating disorders and nonnormative eating behaviors did not show significant prospective associations with weight loss. However, subthreshold binge-eating disorder and loss-of-control eating were significantly prospectively associated with lower HRQOL.

**Meaning:**

The results demonstrate prospective relevance of subthreshold binge-eating disorder and loss-of-control eating for long-term HRQOL outcomes following obesity surgery; postsurgical monitoring may identify patients who need targeted prevention or psychotherapy.

## Teaser

This cohort study of patients who presented for obesity surgery examines the association of presurgical and postsurgical nonnormative eating behaviors and eating disorders with long-term outcomes.

## Introduction

Obesity surgery (OS) is the most efficacious and sustainable intervention for severe obesity (ie, obesity class 3 body mass index [BMI; calculated as weight in kilograms divided by height in meters squared] ≥40 or class 2 BMI ≥35 with obesity-related comorbidities),^1^ an increasingly prevalent health disorder.^2,3,4^ Obesity surgery leads to 20% to 35% total weight loss at 5 to 10 years following laparoscopic Roux-en-Y gastric bypass (RYGB) and laparoscopic sleeve gastrectomy (SG)^5,6^; however, up to 15% of patients experience poor weight loss.^7^ Weight loss is critical for alleviation of the adverse physical (eg, type 2 diabetes)^8,9,10^ and psychological obesity-related sequelae (eg, eating disorders [EDs]),^11^ all associated with quality-of-life improvement.^12,13,14^ Targeting the restriction of the amount of food that patients can consume,^15,16,17,18,19^ OS induces profound changes in eating behavior^20^ with both normative and nonnormative^21^ facets.

Nonnormative eating behaviors (NEBs), including various forms of binge eating or overeating and weight-compensatory behaviors, are common mental health problems in OS,^22,23,24,25,26,27^ with up to one-half of patients being affected.^28^ Overall, approximately 7% to 12% of patients presenting for OS were diagnosed with a full-blown ED,^23^ including binge-eating disorder (BED) or bulimia nervosa (BN), characterized by recurrent objective binge eating (ie, consuming objectively large amounts of food with a sense of loss of control [LOC] over eating) with or without regular compensatory behaviors aimed at preventing weight gain (eg, self-induced vomiting), as defined by the *Diagnostic and Statistical Manual of Mental Disorders, Fifth Edition* (*DSM-5*).^29^ Although both NEBs and EDs were found to be substantially improved following OS,^22,23^ many patients continue to show, develop, or redevelop them over time, with potential detrimental consequences for weight loss and quality of life.^30^ Most consistently, postoperative LOC eating (ie, subjective and objective binge eating, including consumption of objectively or subjectively large amounts of food with a sense of LOC over eating) was concurrently associated with lower weight loss, whereas for objective binge eating or BED, associations with weight loss were less clear.^23,31^ Less and conflicting evidence is available on the prediction of health-related quality of life (HRQOL).^32,33^ In the Longitudinal Assessment of Bariatric Surgery-3 (LABS-3) Psychosocial Study, interview-determined NEBs and preoperative history of EDs were not associated with HRQOL change in patients undergoing RYGB who were followed up for 7 years (N = 104-107).^12,34^

Still, the specific changes in NEBs and EDs and their significance for weight and HRQOL outcomes remain poorly understood. Higher-quality evidence gathered through clinical interview, the standard for ED assessment, is scarce,^28,35,36^ especially at follow-ups longer than 1 year. Whereas only small samples with RYGB have been investigated, evidence on SG, the most commonly performed procedure, is lacking.^12,34,37^ Several EDs, especially those among the *DSM-5* other specified feeding or eating disorders, have gained limited attention, for example, night eating syndrome,^38,39^ atypical anorexia nervosa (AN),^29,40,41^ and purging disorder (Table 1). In addition, the *DSM-5*^29^ has been criticized as unsuitable for postsurgical populations, for example, because alterations in eating behavior associated with the surgically reduced gastric capacity make the uncontrolled ingestion of large amounts of food impossible.^24^ Therefore, the use of LOC eating rather than objective binge eating has been proposed in the diagnosis of BED and BN,^42^ but the prospective relevance needs to be clarified.

**Table 1.** Overview and Definitions of Nonnormative Eating Behaviors and *DSM-5* Eating Disorders

Eating behavior or disorder	Definition
Nonnormative eating behaviors	
Loss-of-control eating	Subjective and/or objective binge eating
Objective binge eating	Consumption of an objectively large amount of food that is definitely larger than what most people would eat given the circumstances, coupled with a sense of loss of control over eating
Subjective binge eating	Consumption of a subjectively but not objectively large amount of food, coupled with a sense of loss of control over eating
Compensatory behaviors	Inappropriate behaviors aimed at preventing weight gain, including self-induced vomiting, laxative misuse, diuretic misuse, extreme dietary restriction, driven exercising, and other compensatory behaviors
Night eating	Nocturnal eating (ie, eating after awakening from sleep) and/or evening eating (ie, excessive food consumption after the evening meal), while being aware of and able to recall but not caused by external circumstances (eg, shift work)
*DSM-5* eating disorders	
Binge-eating disorder^a^	Recurrent objective binge eating at least once a week for 3 mo; no recurrent inappropriate compensatory behavior as in bulimia nervosa, not exclusively during anorexia nervosa; ≥3 of 5 behavioral indicators of binge eating; marked distress
Bulimia nervosa^a^	Recurrent objective binge eating and recurrent compensatory behaviors at least once a week for 3 mo; self-evaluation unduly influenced by body shape and weight; not exclusively during anorexia nervosa
Anorexia nervosa	Restriction of energy intake leading to significantly low weight, which is lower than minimally normal; intense fear of weight gain or becoming fat; disturbance in body weight or shape experience or self-evaluation unduly influenced by body shape and weight
Other specified feeding or eating disorders	Clinically significant eating disorder symptoms without meeting the full criteria of an eating disorder
Binge-eating disorder of low frequency and/or limited duration (subthreshold)^a^	Recurrent objective binge eating less than once a week and/or for <3 mo; all other criteria as for binge-eating disorder
Bulimia nervosa of low frequency and/or limited duration subthreshold (subthreshold)^a^	Recurrent objective binge eating and compensatory behaviors less than once a week and/or for <3 mo; all other criteria as for bulimia nervosa
Atypical anorexia nervosa	Despite significant weight loss, body weight within or above the normal range^b^; all other criteria as for anorexia nervosa
Purging disorder	Recurrent purging behavior (eg, self-induced vomiting, misuse of laxatives, diuretics, or other medications) to influence shape or weight; absence of objective binge eating^c^
Night eating syndrome	Recurrent night eating with awareness and ability to recall and not better explained by external influences; not explained by binge-eating disorder or other mental or physical condition; marked distress^d^

Abbreviation: *DSM-5*, *Diagnostic and Statistical Manual of Mental Disorders, 5th Edition*.^29^

^a^ In an additional exploratory analysis, binge-eating disorder, bulimia nervosa, and subthreshold variants were diagnosed based on loss-of-control eating instead of objective binge eating as required by the *DSM-5*.

^b^ Operationalized as body mass index (calculated as weight in kilograms divided by height in meters squared) of 18.5 to 29.9.

^c^ Operationalized as purging behavior at least once per week for 3 months; fear of weight gain or self-evaluation unduly influenced by shape or weight; fewer than 2 objective binge-eating episodes for 3 months (Pamela Keel, PhD, written communication, September 21, 2020).

^d^ Operationalized as night eating at least once per week for 3 months; no diagnosis of binge-eating disorder, bulimia nervosa, or anorexia nervosa; and marked distress.

This study examined the prevalence and prospective significance of presurgical and postsurgical NEBs and specified EDs, diagnosed through clinical interview according to the *DSM-5*, for weight loss and HRQOL outcomes up to 6 years following RYGB and SG in the largest interview-based longitudinal psychosocial registry of OS to our knowledge. We exploratively addressed the prospective significance of BED and BN and subthreshold variants diagnosed based on LOC eating.

## Methods

### Participants

This cohort study is part of the longitudinal Psychosocial Registry for Obesity Surgery (PRAC), which comprehensively assesses psychosocial aspects in a consecutive OS sample at 6 German treatment centers (eAppendix in the Supplement).^43,44^ With approval of the ethics committees of the 6 German treatment centers, written informed consent was obtained from all patients before study enrollment. This study followed the Strengthening the Reporting of Observational Studies in Epidemiology (STROBE) reporting guideline.^45^

Inclusion criteria were age of 18 years or older and planned OS. Exclusion criteria were lack of German language skills and noncompliance. Study-specific inclusion criteria comprised RYGB or SG as surgical procedures, a complete baseline Eating Disorder Examination (EDE),^46,47^ and enrollment between March 1, 2012, and December 31, 2020. Assessment time points were baseline before surgery (T0) and 6 months (T1) and 1 to 6 years (T2-T7) following surgery.

A total of 1040 adults with planned OS were enrolled in PRAC, of whom 292 were excluded (surgery not received: 164; no RYGB or SG: 60; no complete baseline EDE: 52; and dropout before baseline: 16), leaving a total baseline sample of 748 participants (71.92%) subsequently undergoing RYGB (511 [68.32%]) or SG (237 [31.68%]). For these patients, 2423 follow-up interview assessments were available (T1: 650; T2: 557; T3: 414; T4: 322; T5: 227; T6: 144; and T7: 109). Across follow-up, 93 patients (12.43%) dropped out (T1: 20; T2: 10; T3: 17; T4: 10; T5: 21; T6: 10; and T7: 5) because of withdrawal of consent (77 of 93) or death (16 of 93).

### Measures

Both NEBs and EDs (Table 1) were identified using the EDE, a semistructured expert interview (T0),^46,47^ and its Bariatric Surgery Version (EDE-BSV; T1-T7)^48^ with good interrater reliability,^47,49^ using diagnostic items only (eAppendix in the Supplement). A supplemental night eating module^43^ served to assess night eating and the *DSM-5* criteria of night eating syndrome. Interviews were conducted by trained assessors under regular supervision.

Body weight and height were objectively measured using calibrated instruments. If lacking at follow-up, weight was imputed from subjective body weight (eAppendix in the Supplement). Postoperative weight outcome at T1 to T7 was determined as the percentage of total body weight loss (%TBWL), and HRQOL was determined using the validated Impact of Weight on Quality of Life–Lite^50,51^ questionnaire total score (range, 0-100, with 0 indicating worst and 100 indicating best).

### Statistical Analysis

Data were analyzed from April to November 2021. Prevalence of NEBs and EDs from baseline through follow-up were examined descriptively. Multivariable longitudinal linear mixed-regression models served to analyze associations between NEBs and the outcomes %TBWL and HRQOL at T1 to T7. Concurrent models included fixed effects for NEBs measured at the same time point *t* as the outcomes, whereas prospective models included fixed effects for NEBs measured at the previous time point *t* −1.^52^ Center- and patient-specific random effects accounted for the longitudinal data structure. All models were adjusted for age, sex, baseline weight, surgical procedure (RYGB or SG), and reoperations (yes or no) (fixed effects). The models for postsurgical HRQOL were additionally adjusted for baseline HRQOL. All available data were included without imputing missing values. The same longitudinal mixed-modeling approach was used to examine associations between *DSM-5* EDs and the outcomes %TBWL and HRQOL. Sensitivity analyses addressed the surgical procedure (RYGB subsample analysis), adjustment (total sample analysis without adjustment for age, sex, baseline weight, surgical procedure, and reoperations), and length of follow-up (subsample analysis with ≥2 years of follow-up). All analyses were conducted using R software, version 4.0.5 (R Foundation for Statistical Computing) and applied a 2-tailed α < .05.

## Results

The majority of the 748 patients presenting for OS were middle-aged (mean [SD] age, 46.26 [11.44] years), female (513 [68.58%] women and 235 [31.42%] men), had low school education (506 [83.36%]), were classified as having obesity class 3 (643 [86.42%]), and presented for surgery at Leipzig University Medical Center (599 [80.08%]) (Table 2). During follow-up, a bariatric reoperation was reported in 70 patients (9.36%).

**Table 2.** Sociodemographic and Clinical Characteristics^a^

Characteristic	Total (N = 748)	Gastric bypass (n = 511)	Sleeve gastrectomy (n = 237)
Age, y			
Median (IQR)	47.00 (37.00-55.00)	48.00 (37.00-55.00)	46.00 (37.00-55.00)
Mean (SD)	46.26 (11.44)	46.60 (11.30)	45.54 (11.73)
Sex			
Female	513 (68.58)	367 (71.82)	146 (61.60)
Male	235 (31.42)	144 (28.18)	91 (38.40)
Educational level^b^			
High	101 (16.64)	70 (17.11)	31 (15.66)
Low	506 (83.36)	339 (82.89)	167 (84.34)
Body weight, kg			
Median (IQR)	136.10 (122.00-156.30)	133.20 (119.85-148.75)	152.00 (127.85-174.45)
Mean (SD)	141.28 (28.64)	135.48 (23.66)	153.87 (34.01)
BMI			
Median (IQR)	47.55 (42.20-52.90)	46.60 (41.90-50.90)	51.20 (43.40-57.75)
Mean (SD)	48.38 (8.09)	46.80 (6.49)	51.82 (9.95)
Weight status^c^			
Obesity class 1	11 (1.48)	10 (1.96)	1 (0.43)
Obesity class 2	90 (12.10)	62 (12.18)	28 (11.91)
Obesity class 3	643 (86.42)	437 (85.85)	206 (87.66)
Treatment center			
Leipzig	599 (80.08)	426 (83.37)	173 (73.00)
Other	149 (19.92)	85 (16.63)	64 (27.00)

Abbreviation: BMI, body mass index (calculated as weight in kilograms divided by height in meters squared).

^a^ Data are presented as number (percentage) of patients unless otherwise indicated.

^b^ School education: high indicates 12 years or more; low, less than 12 years.

^c^ Obesity class 1, BMI of 30.0 to 34.9; class 2, BMI of 35.0 to 39.9; and class 3, BMI of 40.0 or greater.

### Weight Loss and HRQOL

Across follow-up, the mean (SD) %TBWL from baseline was 26.70% (9.61%), corresponding to an absolute mean (SD) weight loss of 38.61 (17.07) kg (Figure). Normal weight (BMI, 18.5-24.9) was demonstrated in no patients to 28 of 403 patients (6.95%), overweight (BMI, 25.0-29.9) in 63 of 629 (10.02%) to 94 of 103 (23.33%) patients, and obesity (BMI, ≥30.0) in 201 of 403 (69.73%) to 558 of 629 (88.71%) patients. None of the patients were classified as underweight (BMI, <18.5). Mean (SD) HRQOL improvement from baseline was 35.41 (20.63) percentage points across follow-up.

**Figure.**
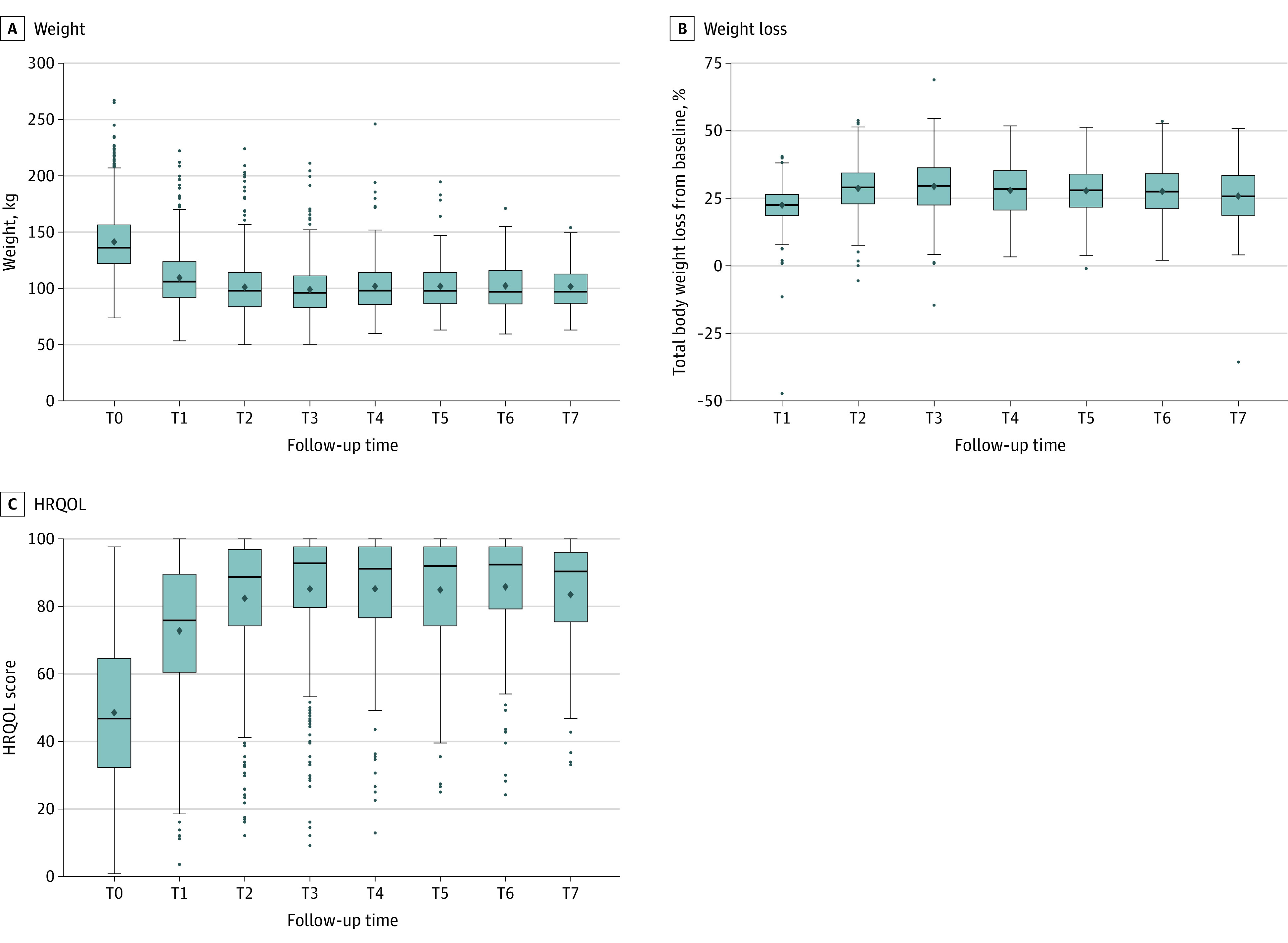
Absolute and Relative Weight Loss and Health-Related Quality of Life (HRQOL) Before Obesity Surgery Through 6 Years of Follow-up in 748 Patients Health-related quality of life was assessed with the Impact of Weight on Quality of Life–Lite total score (range, 0-100, with 0 indicating worst and 100 indicating best).^50,51^ Assessment time points were baseline before surgery (T0) and 6 months (T1) and 1 to 6 years (T2-T7) after surgery. The center points indicate means; upper and lower bounds of the boxes, 25th or 75th percentile; horizontal lines within the boxes, medians; whiskers, minimum and maximum value of the data; and outlying data points, outliers.

### Prevalence of NEBs and EDs

Descriptively, NEBs revealed the highest baseline prevalence for objective and subjective binge eating and extreme dietary restraint and the lowest prevalence for purging behaviors, including self-induced vomiting and laxative and diuretic misuse (and other compensatory behaviors) (Table 3). Except for purging behaviors without marked variations and extreme dietary restraint that decreased overall, NEBs showed an improvement by 6-month follow-up, increasing thereafter at varying degrees. Notably, baseline prevalence was almost reached for subjective binge eating and was exceeded for nocturnal eating and driven exercising.

**Table 3.** Nonnormative Eating Behaviors and Eating Disorders According to the *DSM-5* During 6 Years Following Obesity Surgery

Eating behavior or disorder	No./total No. (%)							
	T0 (baseline)	T1 (6 mo)	T2 (1 y)	T3 (2 y)	T4 (3 y)	T5 (4 y)	T6 (5 y)	T7 (6 y)
**Nonnormative eating behaviors**								
Loss-of-control eating								
Any	168/748 (22.46)	49/649 (7.55)	56/555 (10.09)	48/414 (11.59)	37/322 (11.49)	25/227 (11.01)	16/144 (11.11)	8/109 (7.34)
Objective binge eating	95/747 (12.72)	3/649 (0.46)	4/555 (0.72)	3/414 (0.72)	1/322 (0.31)	6/227 (2.64)	1/144 (0.69)	2/109 (1.83)
Subjective binge eating	87/746 (11.66)	47/649 (7.24)	53/555 (9.55)	45/414 (10.87)	36/322 (11.18)	19/227 (8.37)	15/144 (10.42)	7/109 (6.42)
Night eating								
Any	98/744 (13.17)	35/645 (5.43)	46/557 (8.26)	37/413 (8.96)	34/321 (10.59)	26/227 (11.45)	17/144 (11.81)	14/109 (12.84)
Nocturnal eating	48/747 (6.43)	27/648 (4.17)	27/557 (4.85)	34/414 (8.21)	18/322 (5.59)	17/227 (7.49)	12/144 (8.33)	11/109 (10.09)
Evening eating	64/747 (8.57)	12/647 (1.85)	21/557 (3.77)	9/414 (2.17)	19/321 (5.92)	11/227 (4.85)	8/144 (5.56)	6/109 (5.50)
Compensatory behaviors								
Any	98/693 (14.14)	61/621 (9.82)	47/537 (8.75)	36/397 (9.07)	30/311 (9.65)	16/217 (7.37)	10/135 (7.41)	5/100 (5.00)
Extreme dietary restraint	85/722 (11.77)	51/637 (8.01)	36/549 (6.56)	22/407 (5.41)	24/318 (7.55)	10/220 (4.55)	8/143 (5.59)	3/106 (2.83)
Self-induced vomiting	0/748	3/650 (0.46)	0/556	2/414 (0.48)	0/322	0/227	1/144 (0.69)	0/109
Laxative misuse	2/748 (0.27)	0/650	0/557	1/414 (0.24)	1/322 (0.31)	0/227	0/144	0/109
Diuretic misuse	2/748 (0.27)	0/650	2/556 (0.36)	1/414 (0.24)	0/322	0/227	0/144	0/109
Driven exercising	10/720 (1.39)	8/634 (1.26)	11/546 (2.01)	9/404 (2.23)	7/314 (2.23)	6/224 (2.68)	1/136 (0.74)	1/103 (0.97)
Other compensatory behaviors	3/747 (0.40)	0/650	0/557	2/414 (0.48)	2/322 (0.62)	1/227 (0.44)	0/144	1/109 (0.92)
***DSM-5* eating disorders**								
Binge-eating disorder	26/748 (3.48)	2/649 (0.31)	1/555 (0.18)	0/414	0/322	2/227 (0.88)	0/144	1/109 (0.92)
Bulimia nervosa	4/748 (0.53)	0/649	0/555	0/414	0/322	0/227	0/144	0/109
Anorexia nervosa	0/743	0/644	0/553	0/408	0/322	0/227	0/143	0/107
**Other specified feeding or eating disorder**								
Binge-eating disorder subthreshold	20/748 (2.67)	0/649	1/555 (0.18)	1/414 (0.24)	0/322	1/227 (0.44)	0/144	0/109
Bulimia nervosa subthreshold	11/748 (1.47)	0/649	0/555	1/414 (0.24)	0/322	0/227	0/144	0/109
Atypical anorexia nervosa	0/743	40/644 (6.21)	88/553 (15.91)	61/408 (14.95)	37/322 (11.49)	21/227 (9.25)	12/143 (8.39)	9/107 (8.41)
Purging disorder	4/748 (0.53)	1/650 (0.15)	2/557 (0.36)	3/414 (0.72)	2/322 (0.62)	0/227	0/144	1/109 (0.92)
Night eating syndrome	6/744 (0.81)	3/645 (0.47)	3/557 (0.54)	8/413 (1.94)	4/321 (1.25)	1/227 (0.44)	3/144 (2.08)	3/109 (2.75)
**Eating disorders based on loss-of-control eating^a^**								
Binge-eating disorder	46/748 (6.15)	2/649 (0.31)	6/555 (1.08)	11/414 (2.66)	11/322 (3.42)	4/227 (1.76)	2/144 (1.39)	4/109 (3.67)
Bulimia nervosa	7/748 (0.94)	0/649	3/554 (0.54)	3/414 (0.72)	0/322	0/227	0/144	1/109 (0.92)
Binge-eating disorder subthreshold	41/748 (5.48)	16/649 (2.47)	9/555 (1.62)	9/414 (2.17)	6/322 (1.86)	7/227 (3.08)	3/144 (2.08)	1/109 (0.92)
Bulimia nervosa subthreshold	3/748 (0.40)	0/649	0/555	0/414	0/322	0/227	0/144	0/109

Abbreviation: *DSM-5*, *Diagnostic and Statistical Manual of Mental Disorders, Fifth Edition*.^29^

^a^ Eating disorders were exploratively diagnosed based on loss-of-control eating instead of objective binge eating as required by the *DSM-5*.

The *DSM-5* EDs showed the highest baseline prevalence for BED and subthreshold BED, whereas AN and atypical AN were not detected (Table 3). Most EDs remitted by 6-month follow-up and remained almost absent thereafter (eg, BED, BN, and subthreshold variants). For purging disorder and night eating syndrome, prevalence decreased through 1-year follow-up but exceeded baseline prevalence at multiple time points. Atypical AN, presenting with restricting behaviors only, increased from baseline through 1-year follow-up followed by a decrease, whereas AN was not observed.

### Significance for Weight Loss and HRQOL

Loss-of-control eating at follow-up showed concurrent but not prospective associations with lower %TBWL, with each LOC eating episode associated with a %TBWL decrease of −0.09 (95% CI, −0.14 to −0.04) (Table 4; eTable 1 in the Supplement). This association was attributable to subjective but not objective binge eating. Regarding HRQOL, LOC eating at follow-up showed concurrent and prospective associations with lower HRQOL, with each episode concurrently (−0.14; 95% CI, −0.24 to −0.05 percentage points) or prospectively (−0.10; 95% CI, −0.17 to −0.03 percentage points) associated with an HRQOL decrease. The concurrent association was attributable to both objective and subjective binge eating, whereas only subjective binge eating was prospectively associated with reduced HRQOL. Neither night eating nor compensatory behaviors at follow-up showed significant associations with %TBWL or HRQOL. Baseline NEBs were not significantly associated with %TBWL or HRQOL.

**Table 4.** Percentage Total Body Weight and Health-Related Quality of Life With Nonnormative Eating Behaviors or Eating Disorders According to the *DSM-5* During 6 Years Following Obesity Surgery

Eating behavior or disorder	%TBWL^a^		Health-related quality of life^a^	
	Estimate (95% CI)	Standardized estimate (95% CI)	Estimate (95% CI)	Standardized estimate (95% CI)	
**Nonnormative eating behaviors**					
Loss-of-control eating					
Baseline	0.03 (−0.04 to 0.09)	0.03 (−0.04 to 0.10)	−0.004 (−0.12 to 0.11)	−0.002 (−0.06 to 0.05)	
Concurrent	−0.09 (−0.14 to −0.04)	−0.06 (−0.08 to −0.03)	−0.14 (−0.24 to −0.05)	−0.05 (−0.08 to −0.02)	
Prospective	−0.004 (−0.04 to 0.03)	−0.003 (−0.03 to 0.02)	−0.10 (−0.17 to −0.03)	−0.04 (−0.07 to −0.01)	
Objective binge eating					
Baseline	0.01 (−0.06 to 0.08)	0.01 (−0.06 to 0.08)	0.009 (−0.11 to 0.13)	0.004 (−0.05 to 0.06)	
Concurrent	−0.06 (−0.13 to 0.01)	−0.02 (−0.04 to 0.004)	−0.16 (−0.31 to −0.02)	−0.03 (−0.05 to −0.003)	
Prospective	0.01 (−0.03 to 0.05)	0.009 (−0.02 to 0.04)	−0.06 (0.16 to 0.03)	−0.02 (−0.05 to 0.009)	
Subjective binge eating					
Baseline	0.20 (−0.02 to 0.42)	0.06 (−0.007 to 0.14)	−0.12 (−0.48 to 0.25)	−0.02 (−0.09 to 0.05)	
Concurrent	−0.11 (−0.17 to −0.05)	−0.06 (−0.09 to −0.03)	−0.13 (−0.25 to −0.005)	−0.04 (−0.07 to −0.001)	
Prospective	−0.06 (−0.14 to 0.01)	−0.02 (−0.05 to 0.003)	−0.14 (−0.24 to −0.04)	−0.04 (−0.07 to −0.01)	
Night eating					
Baseline	−0.01 (−0.10 to 0.07)	−0.01 (−0.09 to 0.06)	−0.05 (−0.20 to 0.09)	−0.02 (−0.09 to 0.04)	
Concurrent	0.03 (−0.01 to 0.07)	0.02 (−0.008 to 0.05)	−0.03 (−0.11 to 0.06)	−0.01 (−0.04 to 0.02)	
Prospective	−0.02 (−0.06 to 0.02)	−0.01 (−0.04 to 0.02)	−0.03 (−0.11 to 0.04)	−0.01 (−0.04 to 0.02)	
Compensatory behaviors					
Baseline	−0.14 (−0.33 to 0.05)	−0.05 (−0.12 to 0.02)	0.03 (−0.30 to 0.36)	0.006 (−0.06 to 0.07)	
Concurrent	−0.01 (−0.11 to 0.09)	−0.003 (−0.03 to 0.02)	−0.10 (−0.30 to 0.10)	−0.01 (−0.04 to 0.01)	
Prospective	−0.03 (−0.12 to 0.06)	−0.009 (−0.04 to 0.02)	0.06 (−0.13 to 0.25)	0.008 (−0.02 to 0.03)	
***DSM-5* eating disorders**					
Binge-eating disorder					
Baseline	−0.10 (−3.40 to 3.20)	−0.002 (−0.07 to 0.06)	0.42 (−5.51 to 6.35)	0.004 (−0.06 to 0.06)	
Concurrent	−0.30 (−5.06 to 4.47)	−0.001 (−0.02 to 0.02)	−3.15 (−11.88 to 5.59)	−0.009 (−0.04 to 0.02)	
Prospective	−0.55 (−2.76 to 1.67)	−0.006 (−0.03 to 0.02)	0.36 (−4.42 to 5.14)	0.002 (−0.02 to 0.03)	
Bulimia nervosa^b^					
Baseline	3.34 (−4.28 to 10.96)	0.03 (−0.04 to 0.10)	−7.04 (−21.89 to 7.81)	−0.03 (−0.09 to 0.03)	
Prospective	−0.73 (−6.01 to 4.56)	−0.003 (−0.03 to 0.02)	6.86 (−5.40 to 19.12)	0.01 (−0.01 to 0.04)	
Binge-eating disorder subthreshold					
Baseline	0.43 (−3.54 to 4.41)	0.007 (−0.06 to 0.07)	−2.45 (−9.43 to 4.54)	−0.02 (−0.07 to 0.04)	
Concurrent	−0.40 (−8.78 to 7.99)	−0.002 (−0.03 to 0.03)	−6.80 (−23.18 to 9.59)	−0.01 (−0.05 to 0.02)	
Prospective	−1.00 (−4.09 to 2.10)	−0.009 (−0.04 to 0.02)	−6.51 (−12.69 to −0.34)	−0.03 (−0.06 to −0.002)	
Bulimia nervosa subthreshold					
Baseline	2.45 (−2.91 to 7.81)	0.03 (−0.04 to 0.09)	3.64 (−5.67 to 12.95)	0.02 (−0.04 to 0.08)	
Concurrent	2.62 (−11.39 to 16.63)	0.006 (−0.02 to 0.04)	20.84 (−6.59 to 48.27)	0.02 (−0.008 to 0.06)	
Prospective	−2.53 (−6.64 to 1.57)	−0.02 (−0.04 to 0.01)	−2.22 (−10.50 to 6.07)	−0.007 (−0.03 to 0.02)	
Purging disorder					
Baseline	−1.44 (−9.28 to 6.40)	−0.01 (−0.08 to 0.06)	5.42 (−8.94 to 19.77)	0.02 (−0.04 to 0.09)	
Concurrent	−4.27 (−8.56 to 0.02)	−0.02 (−0.05 to 0.00)	−0.26 (−7.95 to 7.43)	−0.001 (−0.03 to 0.02)	
Prospective	2.02 (−1.76 to 5.81)	0.01 (−0.01 to 0.04)	−6.44 (−19.73 to 6.85)	−0.03 (−0.09 to 0.03)	
Night eating syndrome					
Baseline	0.35 (−6.14 to 6.83)	0.004 (−0.07 to 0.08)	4.23 (−7.25 to 15.70)	0.02 (−0.04 to 0.09)	
Concurrent	1.28 (−1.37 to 3.93)	0.01 (−0.01 to 0.04)	−3.89 (−8.79 to 1.01)	−0.02 (−0.05 to 0.006)	
Prospective	−0.77 (−3.17 to 1.62)	−0.008 (−0.03 to 0.02)	−4.53 (−9.50 to 0.44)	−0.02 (−0.05 to 0.002)	
**Eating disorders based on loss-of-control eating^c^**					
Binge-eating disorder					
Baseline	−0.22 (−2.76 to 2.32)	−0.006 (−0.07 to 0.06)	−3.39 (−7.83 to 1.06)	−0.04 (−0.10 to 0.01)	
Concurrent	−2.52 (−4.49 to −0.54)	−0.03 (−0.06 to −0.007)	−3.10 (−7.00 to 0.81)	−0.02 (−0.05 to 0.006)	
Prospective	−1.83 (−3.34 to −0.32)	−0.03 (−0.06 to −0.005)	−1.94 (−5.01 to 1.13)	−0.02 (−0.04 to 0.01)	
Bulimia nervosa					
Baseline	3.21 (−2.80 to 9.22)	0.04 (−0.03 to 0.11)	−2.83 (−12.69 to 7.02)	−0.02 (−0.08 to 0.05)	
Concurrent	−4.84 (−9.47 to −0.22)	−0.03 (−0.05 to −0.001)	2.22 (−6.88 to 11.33)	0.006 (−0.02 to 0.03)	
Prospective	−5.22 (−8.58 to −1.87)	−0.04 (−0.06 to −0.01)	1.91 (−4.45 to 8.27)	0.008 (−0.02 to 0.03)	
Binge-eating disorder subthreshold					
Baseline	0.01 (−2.78 to 2.80)	0.00 (−0.07 to 0.07)	−0.89 (−5.62 to 3.85)	−0.01 (−0.07 to 0.05)	
Concurrent	0.28 (−1.44 to 2.00)	0.004 (−0.02 to 0.03)	−3.42 (−6.81 to −0.04)	−0.03 (−0.05 to 0.00)	
Prospective	−1.07 (−2.55 to 0.41)	−0.02 (−0.04 to 0.007)	−4.57 (−7.50 to −1.64)	−0.04 (−0.07 to −0.02)	
Bulimia nervosa subthreshold^b^					
Baseline	2.83 (−8.83 to 14.49)	0.02 (−0.06 to 0.09)	−13.06 (−31.44 to 5.32)	−0.04 (−0.10 to 0.02)	
Prospective	−2.42 (−11.89 to 7.05)	−0.009 (−0.04 to 0.03)	12.06 (−6.40 to 30.52)	0.02 (−0.01 to 0.05)	

Abbreviations: *DSM-5, Diagnostic and Statistical Manual of Mental Disorders, Fifth Edition*^29^; %TBWL, percentage of total body weight loss.

^a^ Unstandardized and standardized estimates with 95% CIs from multivariable longitudinal linear mixed-regression models of nonnormative eating behaviors or eating disorder diagnoses in their concurrent and prospective associations with %TBWL and health-related quality of life, assessed with the Impact of Weight on Quality of Life–Lite total score (range, 0-100, with 0 indicating worst and 100 indicating best).^50,51^

^b^ Not detected across follow-up; therefore, concurrent models were not computed, and baseline effect sizes were displayed from prospective models.

^c^ In an exploratory analysis, eating disorders were diagnosed based on loss-of-control eating instead of objective binge eating as required by the *DSM-5*. For comparability, the explorative models additionally included all other *DSM-5* eating disorder diagnoses.

The *DSM-5* EDs at baseline and follow-up had no association with %TBWL (Table 4). Regarding HRQOL, only subthreshold BED at follow-up showed a prospective association with a reduction in HRQOL of −6.51 (95% CI, −12.69 to −0.34) percentage points (eTable 1 in the Supplement), but no further significant baseline, concurrent, or prospective association with HRQOL was found. Because atypical AN was not present at baseline, it was not included in the analyses. Sensitivity analyses for surgical procedure, adjustment, and length of follow-up revealed similar patterns of associations of NEBs and EDs with %TBWL and HRQOL as described above (eTables 2-5 in the Supplement).

### Exploratory Analysis

The exploratory analysis of EDs based on LOC eating showed baseline prevalences of BED, BN, and subthreshold BED that were almost twice as high as those of *DSM-5* equivalents and substantial yet fluctuating reductions at follow-up (Table 3). Both BED and BN at follow-up showed prospective associations, with a change in %TBWL of −1.83% (95% CI, −3.34% to −0.32%) for BED and −5.22% (95% CI, −8.58% to −1.87%) for BN (Table 4; eTable 1 in the Supplement). Regarding HRQOL, only subthreshold BED was prospectively associated with an HRQOL decrease (−4.57 percentage points; 95% CI, −7.50 to −1.64 percentage points).

## Discussion

In this large, prospective, multicenter PRAC study, comprehensive interview-based assessment revealed substantial prevalences of NEBs and specified EDs in patients seeking OS, with improvements occurring from 6 months to 6 years following surgery. For several NEBs and EDs, however, baseline prevalences were reached or exceeded during follow-up. Several NEBs at follow-up, especially LOC eating and subjective binge eating, were concurrently associated with lower %TBWL and were both concurrently and prospectively associated with lower HRQOL. Whereas *DSM-5* ED diagnoses were not associated with %TBWL, only subthreshold BED at follow-up was prospectively associated with lower HRQOL. Other and baseline NEBs and EDs did not reveal any prospective significance. The %TBWL and HRQOL improvements were large.^5,53^

Regarding NEBs, the results corroborate and extend prior research,^22,23,24,25,26,27^ with slight differences likely attributable to varying definitions, operationalizations, and patient characteristics. For example, prevalences of subjective binge eating and LOC eating at baseline and follow-up were somewhat higher than those documented in the LABS-3 Psychosocial Study.^34^ Extreme dietary restraint had a notable baseline prevalence, contributing to a higher prevalence of compensatory behaviors than previously reported without this behavior. Consistently, other compensatory behaviors, especially purging behaviors, were rare. Following postsurgical decreases, baseline prevalence for subjective binge eating was almost reached at follow-up, as previously reported,^34^ whereas for nocturnal eating and driven exercising, they were exceeded at multiple time points. Both nocturnal and evening eating were frequent before surgery, and postsurgical improvements, but not deteriorations, had previously been documented by questionnaire.^34^

Consistent with the literature,^23^ LOC eating was the only NEB with concurrent but not prospective or baseline associations with %TBWL, which was attributable to subjective binge eating. Uniquely, LOC eating, an indicator of distress,^54^ as well as subjective binge eating were both concurrently and prospectively associated with lower HRQOL, and objective binge eating at follow-up revealed a significant concurrent association. Using an obesity-related rather than general measure of HRQOL^13^ may have augmented prospective effect sizes compared with prior research.^34^ In contrast, other NEBs were not associated with HRQOL. Baseline correlates of %TBWL and HRQOL were not identified, underlining a specific relevance of postsurgical LOC and binge-eating behaviors.

Regarding the *DSM-5* EDs, baseline prevalences of BED and BN were lower than previously reported using a different interview schedule and classification system.^12^ Notwithstanding, postsurgical improvement of BED and remission of BN were similarly documented. Both BED and BN at baseline and follow-up were not associated with postoperative weight outcome,^23,31^ and uniquely, only subthreshold BED at follow-up was associated with HRQOL, accounting for a notable prospective reduction in HRQOL by 6.51 percentage points.

The reliance of the *DSM-5*^29^ on objective binge eating in the diagnosis of BED and BN has been criticized as unsuitable for patients undergoing OS, and instead the use of LOC eating has been recommended.^42^ Not surprisingly, our exploratory analysis on EDs diagnosed based on LOC eating involved higher baseline prevalences of (subthreshold) BED and BN. As a novel finding, LOC eating–based BED and BN at follow-up were not only concurrently but also prospectively associated with lower %TBWL, involving a prospective reduction in %TBWL by 1.83% for BED or 5.22% for BN, with the latter figure corresponding to the lower threshold of clinically significant weight loss in behavioral weight loss treatment.^55^ Likewise, LOC eating–based BED at follow-up was not only concurrently but also prospectively associated with a reduction of HRQOL by 4.57 percentage points. Overall, these results speak to an increased prospective significance of BED and BN diagnoses and subthreshold variants in OS if diagnosed based on LOC eating rather than objective binge eating as required by the *DSM-5*.^29^ Of note, the *International Statistical Classification of Diseases and Related Health Problems, 11th Revision *(*ICD-11*),^56^ the official governmental classification scheme in most countries worldwide, stipulates the use of LOC eating in the diagnosis of BED and BN.^57,58^ Because of an increased prospective relevance, our results support this modification for postsurgical populations, although prognostic evidence remains outstanding.

Regarding further *DSM-5* EDs not detected at baseline, atypical AN with restricting behaviors only showed a steep increase through the first year after surgery, which was followed by a decrease through follow-up, associated with aforementioned prevalences of compensatory behaviors. Whereas the definition of a significant weight loss to a normal or higher body weight for diagnosis of atypical AN is generally unclear,^59,60^ for OS it has not been officially defined what constitutes a significant weight loss,^61^ nor is there consensus about a “normal” postsurgical BMI.^24^ In our study, most patients showed a considerable weight loss across follow-up, but most continued to have obesity. A minority of patients reached a BMI in the overweight or normal weight range. For determining a significant weight loss to a normal or higher body weight in the diagnosis of *DSM-5* atypical AN,^29^ we applied a threshold of BMI less than 30.0 because obesity is viewed as clinically relevant.^55^ Anorexia nervosa was not identified in this study because of the absence of underweight, and the *DSM-5* text for AN broadly allowing a body weight below a minimally normal level for age, sex, physical health, and developmental trajectory does not seem to be clearly applicable to OS populations. Using these weight criteria for diagnosis of AN and atypical AN, it needs to be acknowledged that some cases with similar symptoms at higher weight may have been overlooked.^40,62^ Further systematic research is warranted to specify and validate diagnostic criteria for restrictive eating disturbances in OS. Nonnormative eating behaviors and cognitions co-occurring with extreme dietary restriction^40,41,62,63^ could represent additional or alternative criteria to the weight criterion to make diagnoses determinable and comparable before and after surgery.

Baseline prevalence of night eating syndrome obtained by applying full *DSM-5* criteria was lower than previously reported,^38^ and purging disorder, examined for the first time in OS, was rarely observed. Both disorders decreased through the first year following surgery but then recurrently exceeded baseline prevalences. Warranting further investigation is whether behavioral adaptations to OS (eg, restricted meals, food avoidance, and physical activity regimen) foster postsurgical NEBs (eg, purging behaviors, driven exercising, and nocturnal eating) and thereby give rise to these EDs. Previous self-report–based research had highlighted an improvement of night eating syndrome following OS.^38,64,65^

### Strengths and Limitations

Strengths of this interview-based, multicenter study include its large-sample, prospective examination of NEBs and specified EDs following RYGB and SG, the most common surgical procedures worldwide.^66^ Minimal inclusion and exclusion criteria; standardized, internationally well-established assessments, including face-to-face EDE-BSV^46,47,48^ by trained and supervised assessors; statistical control of confounders; and low study dropout across follow-up (12.43%) support low selection, information, and confounding bias and high generalizability to OS populations. This study also has some limitations. With consecutive enrollment, EDE-BSV assessments decreased from 748 to 109 at 6 years, making a sensitivity analysis for SG premature. Notwithstanding, the robustness of results was confirmed for the RYGB subsample.

## Conclusions

Beyond clarification of ED diagnostic criteria in OS, future research is warranted to improve definition and assessment of NEBs.^15,67,68,69^ Because of possible differences in the course and prospective value of NEBs and EDs by surgical procedure,^7,15,17,70^ separate analyses should be conducted for SG at a larger sample size. In addition, factors associated with postsurgical NEBs and EDs deserve clarification beyond their presurgical presentation.^71^ Evidence is widely lacking on long-term trajectories and diagnostic transitions of NEBs and EDs.^30^

Postsurgical EDs often remain undetected and untreated. Our results underline the importance of monitoring not only EDs but also NEBs beyond the first year following surgery, especially because baseline presentations were not associated with %TBWL and HRQOL outcome, which does not provide strong support of EDs as contraindications for OS. Notwithstanding, because of its prospective relevance and as a core behavior of BED and BN according to the *ICD-11*,^56^ postsurgical LOC eating deserves particular attention to identify those in need of additional treatment early and to prevent lesser weight loss and HRQOL.^26,72^

## Notes

Supplement. **eAppendix.** Supplementary Information **eTable 1.** Percent Total Body Weight Loss and Health-related Quality of Life by Presence or Absence of Nonnormative Eating Behaviors and Eating Disorders According to DSM-5 Over 6 Years Following Obesity Surgery for Significant Effects **eTable 2.** Sensitivity Analysis for Surgical Procedure: Nonnormative Eating Behaviors and Eating Disorders According to DSM-5 Over 6 Years Following Roux-en-Y Gastric Bypass **eTable 3.** Sensitivity Analysis for Surgical Procedure: Percent Total Body Weight Loss and Health-related Quality of Life with Nonnormative Eating Behaviors or Eating Disorders According to DSM-5 Over 6 Years Following Roux-en-Y Gastric Bypass **eTable 4.** Sensitivity Analysis for Adjustment: Unadjusted Percent Total Body Weight Loss and Health-related Quality of Life with Nonnormative Eating Behaviors or Eating Disorders According to DSM-5 Over 6 Years Following Obesity Surgery **eTable 5.** Sensitivity Analysis for Length of Follow-up: Percent Total Body Weight Loss and Health-related Quality of Life with Nonnormative Eating Behaviors or Eating Disorders According to DSM-5 Over 6 Years Following Obesity Surgery for Patients with Follow-ups of 2 Years or More
