# Supplementary material for: Nonnormative Eating Behaviors and Eating Disorders and Their Associations With Weight Loss and Quality of Life During 6 Years Following Obesity Surgery
Source: JAMA Netw Open. 2022 Aug 11;5(8):e2226244. doi: 10.1001/jamanetworkopen.2022.26244 (PMC9372790; doi:10.1001/jamanetworkopen.2022.26244)
Supplement: Supplement. — eAppendix. Supplementary Information eTable 1. Percent Total Body Weight Loss and Health-related Quality of Life by Presence or Absence of Nonnormative Eating Behaviors and Eating Disorders According to DSM-5 Over 6 Years Following Obesity Surgery for Significant Effects eTable 2. Sensitivity Analysis for Surgical Procedure: Nonnormative Eating Behaviors and Eating Disorders According to DSM-5 Over 6 Years Following Roux-en-Y Gastric Bypass eTable 3. Sensitivity Analysis for Surgical Procedure: Percent Total Body Weight Loss and Health-related Quality of Life with Nonnormative Eating Behaviors or Eating Disorders According to DSM-5 Over 6 Years Following Roux-en-Y Gastric Bypass eTable 4. Sensitivity Analysis for Adjustment: Unadjusted Percent Total Body Weight Loss and Health-related Quality of Life with Nonnormative Eating Behaviors or Eating Disorders According to DSM-5 Over 6 Years Following Obesity Surgery eTable 5. Sensitivity Analysis for Length of Follow-up: Percent Total Body Weight Loss and Health-related Quality of Life with Nonnormative Eating Behaviors or Eating Disorders According to DSM-5 Over 6 Years Following Obesity Surgery for Patients with Follow-ups of 2 Years or More [file jamanetwopen-e2226244-s001.pdf]

Supplementary Online Content

Hilbert A, Staerk C, Strömer A, et al. Nonnormative eating behaviors and eating disorders and their associations with weight loss and quality of life during 6 years following obesity surgery. *JAMA Netw Open*. 2022;5(8):e2226244. doi:10.1001/jamanetworkopen.2022.26244

eAppendix. Supplementary Information
eTable 1. Percent Total Body Weight Loss and Health-related Quality of Life by Presence or Absence of Nonnormative Eating Behaviors and Eating Disorders According to DSM-5 Over 6 Years Following Obesity Surgery for Significant Effects
eTable 2. Sensitivity Analysis for Surgical Procedure: Nonnormative Eating Behaviors and Eating Disorders According to DSM-5 Over 6 Years Following Roux-en-Y Gastric Bypass
eTable 3. Sensitivity Analysis for Surgical Procedure: Percent Total Body Weight Loss and Health-related Quality of Life with Nonnormative Eating Behaviors or Eating Disorders According to DSM-5 Over 6 Years Following Roux-en-Y Gastric Bypass
eTable 4. Sensitivity Analysis for Adjustment: Unadjusted Percent Total Body Weight Loss and Health-related Quality of Life with Nonnormative Eating Behaviors or Eating Disorders According to DSM-5 Over 6 Years Following Obesity Surgery
eTable 5. Sensitivity Analysis for Length of Follow-up: Percent Total Body Weight Loss and Health-related Quality of Life with Nonnormative Eating Behaviors or Eating Disorders According to DSM-5 Over 6 Years Following Obesity Surgery for Patients with Follow-ups of 2 Years or More

This supplementary material has been provided by the authors to give readers additional information about their work.

© 2022 Hilbert A et al. *JAMA Network Open.*

eAppendix. Supplementary Information

Overlap with Previous Studies From the Psychosocial Registry for Obesity Surgery

The manuscript is based on the prospective multicenter Psychosocial Registry for Obesity Surgery (PRAC) study aiming to comprehensively assess psychosocial aspects in a consecutive obesity surgery sample prior to surgery and (bi-)annually over 8 years of follow-up. Previous publications used the baseline data only,[1-4] with one exception, a latent profile analysis examining temperament profiles in their association with eating disorder and general psychopathology as well as weight loss over 3 years following obesity surgery.[5] When compared to the current study, the previous study is thematically (temperament profiles vs. nonnormative eating behaviors and eating disorders) and methodologically different (latent profile analyses, multivariate analyses of variance vs. multivariable longitudinal linear mixed regression models), included less than half of the patients (317 vs. 748 patients) and a shorter follow-up timeframe (3 vs. 6 years). Thus, the current study is unique in its focus, methodology, and presentation of PRAC data.

Measures

Nonnormative eating behaviors and eating disorders were identified using the Eating Disorder Examination, a semi-structured expert interview (T0),[6,7] and its Bariatric Surgery Version (EDE-BSV; T1-T7),[8] applying diagnostic items only. The EDE(-BSV) was used for assessment of objective and subjective binge-eating episodes, with both of these representing loss of control eating episodes, and for assessment of compensatory behaviors (i.e., self-induced vomiting, laxative misuse, diuretic misuse, extreme dietary restriction, driven exercising, and other extreme weight-control behaviors). According to the Diagnostic and Statistical Manual of Mental Disorders 5th Edition (DSM-5)[9] diagnoses of binge-eating disorder (BED), bulimia nervosa (BN), and anorexia nervosa, and the Other Specified Feeding or Eating Disorder diagnoses of BED or BN of low frequency and/or limited duration (termed "subthreshold"), atypical anorexia nervosa, and purging disorder were derived. Additional interview items covered the full DSM-5 criteria of night eating syndrome, including nocturnal eating (i.e., episodes of eating after awakening from sleep) and evening eating (i.e., eating after the evening meal), external influences on them, awareness and ability to recall them as well as associated distress.[10] In an exploratory analysis, eating disorders were diagnosed based on loss of control eating instead of objective binge eating as required by the DSM-5.[9]

Nonnormative eating behaviors were assessed for the past 28 days and dichotomized to any nonnormative eating behaviors. Eating disorder diagnoses were determined for the past 3 months. EDE(-BSV) interviews were conducted by trained assessors (i.e., BSc or MSc psychology students, MD students) under regular supervision by a BSc-, MSc-, or PhD-level psychologist.

Body weight and height were objectively measured using calibrated instruments. If objective measurements were unavailable at follow-up, but subjective weight was assessed, objective body weights were imputed from subjective weights based on simple linear regression models. Postoperative weight outcome was determined as the percentage of total body weight loss (%TBWL) at T1-T7, calculated as 100-(100*weight at follow-up/weight at baseline).

Health-related quality of life was determined using the Impact of Weight on Quality of Life-Lite[11,12] questionnaire (31 items; 1=*never true* to 5=*always true*). The IWQOL-Lite has good validity and excellent reliability. In this study, the total sum score was used (Cronbach's α=.95, 95% CI .95-.99; McDonald's ω total=.97, 95% CI .97-1.00), standardized to 0-100, with higher scores indicating better health-related quality of life.

eReferences

1. Baldofski S, Tigges W, Herbig B, et al. Nonnormative eating behavior and psychopathology in prebariatric patients with binge-eating disorder and night eating syndrome. *Surg Obes Relat Dis*. 2015;11(3):621-626. doi:10.1016/j.soard.2014.09.018
2. Hübner C, Baldofski S, Zenger M, et al. Influences of general self-efficacy and weight bias internalization on physical activity in bariatric surgery candidates. *Surg Obes Relat Dis*. 2015;11(6):1371-1376. doi:10.1016/j.soard.2014.11.013
3. Baldofski S, Rudolph A, Tigges W, et al. Weight bias internalization, emotion dysregulation, and non-normative eating behaviors in prebariatric patients. *Int J Eat Disord*. 2016;49(2):180-185. doi:10.1002/eat.22484
4. Schäfer L, Hübner C, Carus T, et al. Identifying prebariatric subtypes based on temperament traits, emotion dysregulation, and disinhibited eating: A latent profile analysis. *Int J Eat Disord*. 2017;50(10):1172-1182. doi:10.1002/eat.22760
5. Schäfer L, Hübner C, Carus T, et al. Pre- and postbariatric subtypes and their predictive value for health-related outcomes measured 3 years after surgery. *Obes Surg*. 2019;29(1):230-238. doi:10.1007/s11695-018-3524-1
6. Fairburn CG, Cooper Z, O'Connor M. Eating Disorder Examination (17.0D). New York. NY: Guilford Press; 2014.

7. Hilbert A, Tuschen-Caffier B. Eating Disorder Examination: Deutschsprachige Übersetzung (Bd. 01, 2. Auflage) [Eating Disorder Examination: German Version (2nd ed.)]. Tübingen, Germany: dgvt-Verlag.
8. de Zwaan M, Hilbert A, Swan-Kremeier L, et al. Comprehensive interview assessment of eating behavior 18-35 months after gastric bypass surgery for morbid obesity. *Surg Obes Relat Dis*. 2010;6(1):79-85. doi:10.1016/j.soard.2009.08.011
9. American Psychiatric Association (APA). Diagnostic and Statistical Manual of Mental Disorders, 5th ed. Arlington, VA: American Psychiatric Publishing; 2013.
10. Baldofski S, Tigges W, Herbig B, et al. Nonnormative eating behavior and psychopathology in prebariatric patients with binge-eating disorder and night eating syndrome. *Surg Obes Relat Dis*. 2015;11(3):621-626. doi:10.1016/j.soard.2014.09.018
11. Kolotkin RL, Crosby RD, Kosloski KD, Williams GR. Development of a brief measure to assess quality of life in obesity. *Obes Res*. 2001;9(2):102-111. doi:10.1038/oby.2001.13
12. Mueller A, Holzapfel C, Hauner H, et al. Psychometric evaluation of the German version of the impact of weight on Quality of Life-Lite (IWQOL-Lite) questionnaire. *Exp Clin Endocrinol Diabetes*. 2011;119(2):69-74. doi:10.1055/s-0030-1261922

© 2022 Hilbert A et al. *JAMA Network Open.*

eTable 1. Percent Total Body Weight Loss and Health-related Quality of Life by Presence or Absence of Nonnormative Eating Behaviors and Eating Disorders According to DSM-5 Over 6 Years Following Obesity Surgery for Significant Effects

		Percent total body weight loss		Health-related quality of life	
		Present	Absent	Present	Absent
		Mean (SD)	Mean (SD)	Mean (SD)	Mean (SD)
**Nonnormative eating behavior**					
Loss of control eating	Concurrent	26.85 (9.69)	27.23 (9.52)	76.05 (21.25)	84.22 (16.65)
	Prospective	NA	NA	75.57 (21.49)	83.94 (16.66)
Objective binge eating	Concurrent	NA	NA	77.03 (20.26)	83.18 (17.25)
Subjective binge eating	Concurrent	25.68 (8.93)	27.28 (9.40)	75.14 (21.22)	83.87 (16.71)
	Prospective	NA	NA	77.66 (20.15)	83.86 (16.92)
**DSM-5 Eating disorders: Other specified feeding or eating disorders**					
Binge-eating disorder subthreshold	Prospective	NA	NA	71.18 (21.69)	83.27 (17.39)
**Eating disorders based on loss of control eating[a]**					
Binge-eating disorder	Concurrent	26.77 (10.73)	27.12 (9.52)	NA	NA
	Prospective	26.40 (8.35)	27.50 (9.62)	NA	NA
Bulimia nervosa	Concurrent	22.96 (7.84)	27.18 (9.54)	NA	NA
	Prospective	24.24 (7.22)	27.49 (9.66)	NA	NA
Binge-eating disorder	Concurrent	NA	NA	75.96 (21.69)	83.29 (17.19)
subthreshold	Prospective	NA	NA	77.48 (16.70)	83.41 (17.33)

Abbreviations: DSM-5, Diagnostic and Statistical Manual of Mental Disorders 5th Edition.[9] NA, not analyzed due to nonsignificant effects in multivariable longitudinal linear mixed regression analyses (*P*<.05).

[a]Exploratively, eating disorders were diagnosed based on loss of control eating instead of objective binge eating as required by the DSM-5.

© 2022 Hilbert A et al. *JAMA Network Open.*

eTable 2. Sensitivity Analysis for Surgical Procedure: Nonnormative Eating Behaviors and Eating Disorders According to DSM-5 Over 6 Years Following Roux-en-Y Gastric Bypass

	Baseline	6 months	1 year	2 years	3 years	4 years	5 years	6 years
	T0	T1	T2	T3	T4	T5	T6	T7
	No./N (%)	No./N (%)	No./N (%)	No./N (%)	No./N (%)	No./N (%)	No./N (%)	No./N (%)
**Nonnormative eating behaviors**								
**Loss of control eating**	108/511 (21.14)	41/470 (8.72)	41/409 (10.02)	40/323 (12.38)	24/253 (9.49)	20/174 (11.49)	14/118 (11.86)	8/89 (8.99)
Objective binge eating	55/510 (10.78)	3/470 (0.64)	4/409 (0.98)	3/323 (0.93)	2/253 (0.79)	6/174 (3.45)	1/118 (0.85)	3/89 (3.37)
Subjective binge eating	61/509 (11.98)	39/470 (8.30)	38/409 (9.29)	37/323 (11.46)	22/253 (8.70)	14/174 (8.05)	13/118 (11.02)	6/89 (6.74)
**Night eating**	69/509 (13.56)	28/467 (6.00)	32/410 (7.80)	30/322 (9.32)	28/252 (11.11)	20/174 (11.49)	15/117 (12.82)	14/88 (15.91)
Nocturnal eating	35/510 (6.86)	22/470 (4.68)	19/410 (4.63)	28/323 (8.67)	17/253 (6.72)	17/174 (9.77)	11/117 (9.40)	13/89 (14.61)
Evening eating	46/510 (9.02)	12/469 (2.56)	16/410 (3.90)	6/323 (1.86)	15/252 (5.95)	5/174 (2.87)	7/118 (5.93)	6/89 (6.74)
**Compensatory behaviors**	67/471 (14.23)	43/451 (9.53)	37/397 (9.32)	28/308 (9.09)	17/245 (6.94)	9/164 (5.49)	6/111 (5.41)	3/82 (3.66)
Extreme dietary restraint	58/491 (11.81)	37/462 (8.01)	31/406 (7.64)	17/316 (5.38)	15/250 (6.00)	6/166 (3.61)	5/117 (4.27)	2/86 (2.33)
Self-induced vomiting	0/511 (0.00)	1/471 (0.21)	0/410 (0.00)	0/323 (0.00)	0/253 (0.00)	0/174 (0.00)	1/118 (0.85)	0/89 (0.00)
Laxative misuse	2/511 (0.39)	0/471 (0.00)	0/410 (0.00)	0/323 (0.00)	0/253 (0.00)	0/174 (0.00)	0/118 (0.00)	0/89 (0.00)
Diuretic misuse	1/511 (0.20)	0/471 (0.00)	2/409 (0.49)	1/323 (0.31)	0/253 (0.00)	0/174 (0.00)	0/118 (0.00)	0/89 (0.00)
Driven exercising	6/491 (1.22)	6/460 (1.30)	6/402 (1.49)	9/315 (2.86)	2/248 (0.81)	3/172 (1.74)	0/112 (0.00)	0/85 (0.00)
Other compensatory behaviors	2/511 (0.39)	0/471 (0.00)	0/410 (0.00)	2/323 (0.62)	1/253 (0.40)	1/174 (0.57)	0/118 (0.00)	1/89 (1.12)
**DSM-5 Eating disorders**								
**Binge-eating disorder**	13/511 (2.54)	2/470 (0.43)	1/409 (0.24)	0/323 (0.00)	0/253 (0.00)	2/174 (1.15)	1/118 (0.85)	1/89 (1.12)
**Bulimia nervosa**	2/511 (0.39)	0/470 (0.00)	0/409 (0.00)	0/323 (0.00)	0/253 (0.00)	0/174 (0.00)	0/118 (0.00)	0/89 (0.00)

© 2022 Hilbert A et al. *JAMA Network Open.*

	Baseline	6 months	1 year	2 years	3 years	4 years	5 years	6 years
	**T0**	**T1**	**T2**	**T3**	**T4**	**T5**	**T6**	**T7**
	**No./N (%)**	**No./N (%)**	**No./N (%)**	**No./N (%)**	**No./N (%)**	**No./N (%)**	**No./N (%)**	**No./N (%)**
**Anorexia nervosa**	0/508 (0.00)	0/468 (0.00)	0/407 (0.00)	0/318 (0.00)	0/253 (0.00)	0/173 (0.00)	0/117 (0.00)	0/88 (0.00)
**Other specified feeding or eating disorders**								
Binge-eating disorder subthreshold	11/511 (2.15)	0/470 (0.00)	1/409 (0.24)	0/323 (0.00)	0/253 (0.00)	1/174 (0.57)	0/118 (0.00)	0/89 (0.00)
Bulimia nervosa subthreshold	6/511 (1.17)	0/470 (0.00)	0/409 (0.00)	0/323 (0.00)	0/253 (0.00)	0/174 (0.00)	0/118 (0.00)	0/89 (0.00)
Atypical anorexia nervosa	0/508 (0.00)	36/468 (7.69)	71/407 (17.44)	56/318 (17.61)	35/253 (13.83)	17/173 (9.83)	13/117 (11.11)	8/88 (9.09)
Purging disorder	3/511 (0.59)	0/471 (0.00)	2/410 (0.49)	1/323 (0.31)	1/253 (0.40)	0/174 (0.00)	0/118 (0.00)	1/89 (1.12)
Night eating syndrome	4/509 (0.79)	3/467 (0.64)	4/410 (0.98)	8/322 (2.48)	4/252 (1.59)	0/174 (0.00)	3/117 (2.56)	3/88 (3.41)
**Eating disorders based on loss of control eating[a]**								
**Binge-eating disorder**	25/511 (4.89)	2/470 (0.43)	5/409 (1.22)	9/323 (2.79)	9/253 (3.56)	2/174 (1.15)	5/118 (4.24)	5/89 (5.62)
**Bulimia nervosa**	3/511 (0.59)	1/470 (0.21)	3/408 (0.74)	3/323 (0.93)	0/253 (0.00)	0/174 (0.00)	0/118 (0.00)	0/89 (0.00)
**Binge-eating disorder subthreshold**	28/511 (5.48)	13/470 (2.77)	8/409 (1.96)	7/323 (2.17)	3/253 (1.19)	7/174 (4.02)	1/118 (0.85)	1/89 (1.12)
**Bulimia nervosa subthreshold**	2/511 (0.39)	0/470 (0.00)	0/409 (0.00)	0/323 (0.00)	0/253 (0.00)	0/174 (0.00)	0/118 (0.00)	0/89 (0.00)

Abbreviations: DSM-5, Diagnostic and Statistical Manual of Mental Disorders 5th Edition.[9]

[a]Exploratively, eating disorders were diagnosed based on loss of control eating instead of objective binge eating as required by the DSM-5.

© 2022 Hilbert A et al. *JAMA Network Open.*

eTable 3. Sensitivity Analysis for Surgical Procedure: Percent Total Body Weight Loss and Health-related Quality of Life with Nonnormative Eating Behaviors or Eating Disorders According to DSM-5 Over 6 Years Following Roux-en-Y Gastric Bypass

		Percent total body weight loss[a]		Health-related quality of life[a]	
		Estimate (95% CI)	Std. estimate (95% CI)	Estimate (95% CI)	Std. estimate (95% CI)
**Nonnormative eating behaviors**					
Loss of control eating	Baseline	0.212 (0.016 to 0.408)	0.096 (0.007 to 0.185)	-0.046 (-0.360 to 0.268)	-0.012 (-0.096 to 0.071)
	Concurrent	-0.088 (-0.138 to -0.038)	-0.058 (-0.091 to -0.025)	-0.122 (-0.218 to -0.027)	-0.048 (-0.085 to -0.011)
	Prospective	-0.013 (-0.070 to 0.043)	-0.007 (-0.035 to 0.021)	-0.153 (-0.242 to -0.064)	-0.058 (-0.092 to -0.024)
Objective binge eating	Baseline	0.234 (-0.047 to 0.516)	0.078 (-0.016 to 0.172)	0.128 (-0.371 to 0.627)	0.021 (-0.062 to 0.105)
	Concurrent	-0.064 (-0.134 to 0.006)	-0.025 (-0.052 to 0.002)	-0.160 (-0.296 to -0.024)	-0.036 (-0.066 to -0.005)
	Prospective	0.024 (-0.042 to 0.089)	0.010 (-0.018 to 0.037)	-0.218 (-0.347 to -0.089)	-0.050 (-0.079 to -0.020)
Subjective binge eating	Baseline	0.203 (-0.077 to 0.482)	0.063 (-0.024 to 0.149)	-0.164 (-0.589 to 0.261)	-0.031 (-0.112 to 0.050)
	Concurrent	-0.115 (-0.184 to -0.046)	-0.061 (-0.098 to -0.024)	-0.087 (-0.215 to 0.042)	-0.028 (-0.069 to 0.013)
	Prospective	-0.116 (-0.220 to -0.013)	-0.031 (-0.059 to -0.004)	-0.099 (-0.216 to 0.019)	-0.030 (-0.066 to 0.006)
Night eating	Baseline	-0.072 (-0.176 to 0.032)	-0.061 (-0.148 to 0.027)	-0.071 (-0.237 to 0.095)	-0.033 (-0.111 to 0.045)
	Concurrent	0.015 (-0.032 to 0.062)	0.011 (-0.024 to 0.047)	-0.071 (-0.161 to 0.019)	-0.030 (-0.069 to 0.008)
	Prospective	-0.020 (-0.067 to 0.026)	-0.014 (-0.045 to 0.018)	-0.039 (-0.123 to 0.044)	-0.017 (-0.052 to 0.019)
Compensatory behaviors	Baseline	-0.040 (-0.301 to 0.220)	-0.011 (-0.083 to 0.061)	-0.108 (-0.603 to 0.386)	-0.016 (-0.089 to 0.057)
	Concurrent	-0.039 (-0.151 to 0.073)	-0.010 (-0.039 to 0.019)	0.096 (-0.138 to 0.331)	0.013 (-0.019 to 0.045)
	Prospective	-0.008 (-0.128 to 0.111)	-0.002 (-0.034 to 0.030)	0.017 (-0.235 to 0.268)	0.002 (-0.030 to 0.034)
**DSM-5 Eating disorders**					
Binge-eating disorder	Baseline	-0.015 (-4.664 to 4.633)	0.000 (-0.074 to 0.073)	-0.233 (-8.685 to 8.220)	-0.002 (-0.077 to 0.073)
	Concurrent	-0.422 (-5.035 to 4.192)	-0.002 (-0.029 to 0.025)	-2.973 (-11.239 to 5.293)	-0.011 (-0.041 to 0.019)
	Prospective	-0.310 (-3.164 to 2.544)	-0.003 (-0.034 to 0.027)	-5.770 (-11.716 to 0.176)	-0.030 (-0.061 to 0.001)
Bulimia nervosa[b]	Baseline	2.855 (-7.921 to 13.632)	0.022 (-0.062 to 0.106)	2.006 (-15.721 to 19.733)	0.008 (-0.064 to 0.080)
	Prospective	0.436 (-6.822 to 7.694)	0.002 (-0.025 to 0.028)	-3.393 (-17.669 to 10.883)	-0.007 (-0.036 to 0.022)
Binge-eating disorder subthreshold	Baseline	-2.822 (-8.095 to 2.452)	-0.041 (-0.119 to 0.036)	-3.042 (-11.148 to 5.065)	-0.025 (-0.093 to 0.042)
	Concurrent	-0.452 (-8.592 to 7.688)	-0.002 (-0.032 to 0.028)	-6.687 (-22.213 to 8.838)	-0.014 (-0.047 to 0.019)
	Prospective	-1.251 (-5.167 to 2.664)	-0.010 (-0.040 to 0.021)	-7.754 (-14.554 to -0.954)	-0.037 (-0.069 to -0.005)
Bulimia nervosa subthreshold[b]	Baseline	-0.656 (-8.479 to 7.166)	-0.007 (-0.088 to 0.074)	8.291 (-6.710 to 23.291)	0.039 (-0.032 to 0.111)
	Prospective	-0.717 (-6.107 to 4.673)	-0.004 (-0.032 to 0.024)	9.009 (-3.282 to 21.299)	0.022 (-0.008 to 0.053)
Purging disorder	Baseline	3.660 (-5.404 to 12.725)	0.032 (-0.047 to 0.110)	0.478 (-16.355 to 17.310)	0.002 (-0.077 to 0.081)
	Concurrent	-4.873 (-10.030 to 0.283)	-0.026 (-0.053 to 0.001)	3.816 (-4.884 to 12.517)	0.013 (-0.016 to 0.042)
	Prospective	3.738 (-1.373 to 8.848)	0.022 (-0.008 to 0.051)	2.651 (-6.161 to 11.463)	0.009 (-0.020 to 0.037)
Night eating syndrome	Baseline	-2.416 (-10.114 to 5.283)	-0.029 (-0.121 to 0.063)	3.798 (-8.394 to 15.990)	0.024 (-0.054 to 0.103)
	Concurrent	1.777 (-0.981 to 4.534)	0.020 (-0.011 to 0.052)	-3.831 (-8.478 to 0.817)	-0.027 (-0.059 to 0.006)

© 2022 Hilbert A et al. *JAMA Network Open.*

		Percent total body weight loss[a]		Health-related quality of life[a]	
		Estimate (95% CI)	Std. estimate (95% CI)	Estimate (95% CI)	Std. estimate (95% CI)
	Prospective	-0.406 (-2.929 to 2.117)	-0.005 (-0.034 to 0.024)	-4.661 (-9.429 to 0.108)	-0.029 (-0.059 to 0.001)
**Eating disorders based on loss of control eating[c]**					
Binge-eating disorder	Baseline	-0.661 (-4.030 to 2.709)	-0.014 (-0.086 to 0.058)	-5.954 (-11.641 to -0.267)	-0.071 (-0.140 to -0.003)
	Concurrent	-2.554 (-4.671 to -0.437)	-0.034 (-0.062 to -0.006)	-2.948 (-7.007 to 1.111)	-0.023 (-0.056 to 0.009)
	Prospective	-2.271 (-4.049 to -0.492)	-0.039 (-0.069 to -0.008)	-2.973 (-6.504 to 0.558)	-0.027 (-0.060 to 0.005)
Bulimia nervosa	Baseline	1.468 (-7.825 to 10.760)	0.014 (-0.074 to 0.101)	5.514 (-9.464 to 20.493)	0.029 (-0.051 to 0.109)
	Concurrent	-1.548 (-6.601 to 3.505)	-0.009 (-0.039 to 0.021)	2.112 (-6.582 to 10.806)	0.008 (-0.024 to 0.039)
	Prospective	-4.609 (-8.704 to -0.514)	-0.032 (-0.060 to -0.004)	-3.437 (-10.879 to 4.006)	-0.014 (-0.044 to 0.016)
Binge-eating disorder	Baseline	-1.382 (-4.710 to 1.947)	-0.033 (-0.114 to 0.047)	0.259 (-4.989 to 5.507)	0.003 (-0.066 to 0.073)
subthreshold	Concurrent	0.757 (-1.162 to 2.677)	0.012 (-0.018 to 0.041)	-3.580 (-7.158 to -0.002)	-0.032 (-0.063 to 0.000)
	Prospective	-0.742 (-2.350 to 0.866)	-0.014 (-0.043 to 0.016)	-3.973 (-7.054 to -0.893)	-0.041 (-0.073 to -0.009)
Bulimia nervosa	Baseline	0.275 (-12.796 to 13.345)	0.002 (-0.088 to 0.092)	-4.688 (-24.648 to 15.272)	-0.018 (-0.094 to 0.058)
subthreshold[b]	Prospective	-4.080 (-13.609 to 5.448)	-0.015 (-0.050 to 0.020)	14.992 (-4.412 to 34.396)	0.022 (-0.006 to 0.050)

Abbreviations: DSM-5, Diagnostic and Statistical Manual of Mental Disorders 5th Edition.[9]

[a]Unstandardized and standardized estimates with 95% confidence intervals from multivariable longitudinal linear mixed regression models of nonnormative eating behaviors or eating disorder diagnoses in their concurrent and prospective associations with percent total body weight loss and health-related quality of life, assessed with the Impact of Weight on Quality of Life-Lite total score (0=*worst* to 100=*best*).[11,12]

[b]Not detected across follow-up, therefore concurrent models were not computed, and baseline effects were displayed from prospective models.

[c]In an exploratory analysis, eating disorders were diagnosed based on loss of control eating instead of objective binge eating as required by the DSM-5. For comparability, the explorative models additionally included all other DSM-5 eating disorder diagnoses.

© 2022 Hilbert A et al. *JAMA Network Open.*

eTable 4. Sensitivity Analysis for Adjustment: Unadjusted Percent Total Body Weight Loss and Health-related Quality of Life with Nonnormative Eating Behaviors or Eating Disorders According to DSM-5 Over 6 Years Following Obesity Surgery

		Percent total body weight loss[a]		Health-related quality of life[a]	
		**Estimate (95% CI)**	**Std. estimate (95% CI)**	**Estimate (95% CI)**	**Std. estimate (95% CI)[a]**
**Nonnormative eating behaviors**					
Loss of control eating	Baseline	0.018 (-0.049 to 0.086)	0.019 (-0.052 to 0.091)	-0.150 (-0.293 to -0.006)	-0.070 (-0.137 to -0.003)
	Concurrent	-0.092 (-0.137 to -0.046)	-0.056 (-0.084 to -0.028)	-0.149 (-0.246 to -0.051)	-0.047 (-0.078 to -0.016)
	Prospective	-0.004 (-0.039 to 0.032)	-0.003 (-0.029 to 0.024)	-0.106 (-0.182 to -0.031)	-0.042 (-0.072 to -0.013)
Objective binge	Baseline	0.002 (-0.068 to 0.073)	0.002 (-0.069 to 0.073)	-0.148 (-0.298 to 0.003)	-0.062 (-0.126 to 0.001)
eating	Concurrent	-0.063 (-0.136 to 0.010)	-0.021 (-0.045 to 0.003)	-0.168 (-0.315 to -0.021)	-0.029 (-0.054 to -0.004)
	Prospective	0.014 (-0.026 to 0.054)	0.010 (-0.017 to 0.036)	-0.074 (-0.171 to 0.023)	-0.022 (-0.050 to 0.007)
Subjective binge	Baseline	0.205 (-0.023 to 0.433)	0.065 (-0.007 to 0.138)	-0.158 (-0.623 to 0.308)	-0.029 (-0.115 to 0.057)
eating	Concurrent	-0.113 (-0.170 to -0.056)	-0.059 (-0.088 to -0.029)	-0.138 (-0.264 to -0.012)	-0.037 (-0.070 to -0.003)
	Prospective	-0.066 (-0.137 to 0.006)	-0.023 (-0.047 to 0.002)	-0.146 (-0.252 to -0.040)	-0.040 (-0.068 to -0.011)
Night eating	Baseline	0.000 (-0.087 to 0.086)	0.000 (-0.076 to 0.075)	-0.116 (-0.298 to 0.066)	-0.051 (-0.132 to 0.029)
	Concurrent	0.030 (-0.012 to 0.073)	0.021 (-0.009 to 0.051)	0.002 (-0.085 to 0.089)	0.001 (-0.031 to 0.033)
	Prospective	-0.023 (-0.066 to 0.021)	-0.015 (-0.044 to 0.014)	-0.018 (-0.097 to 0.061)	-0.007 (-0.037 to 0.023)
Compensatory	Baseline	-0.108 (-0.299 to 0.083)	-0.041 (-0.113 to 0.031)	-0.111 (-0.538 to 0.316)	-0.020 (-0.098 to 0.057)
behaviors	Concurrent	-0.004 (-0.103 to 0.095)	-0.001 (-0.027 to 0.025)	-0.133 (-0.328 to 0.061)	-0.019 (-0.047 to 0.009)
	Prospective	-0.035 (-0.126 to 0.057)	-0.010 (-0.037 to 0.017)	-0.020 (-0.209 to 0.170)	-0.003 (-0.029 to 0.024)
**DSM-5 Eating disorders**					
Binge-eating disorder	Baseline	0.447 (-2.919 to 3.813)	0.009 (-0.057 to 0.075)	-7.010 (-14.335 to 0.315)	-0.070 (-0.144 to 0.003)
	Concurrent	-0.278 (-5.048 to 4.492)	-0.001 (-0.025 to 0.022)	-3.949 (-12.967 to 5.069)	-0.011 (-0.037 to 0.014)
	Prospective	-0.574 (-2.794 to 1.646)	-0.007 (-0.033 to 0.019)	0.926 (-3.866 to 5.718)	0.005 (-0.021 to 0.031)
Bulimia nervosa[b]	Baseline	3.214 (-4.579 to 11.008)	0.031 (-0.044 to 0.105)	-11.376 (-30.559 to 7.807)	-0.048 (-0.128 to 0.033)
	Prospective	-0.803 (-6.091 to 4.485)	-0.004 (-0.027 to 0.020)	6.782 (-5.690 to 19.254)	0.013 (-0.011 to 0.038)
Binge-eating disorder	Baseline	-0.042 (-4.115 to 4.030)	-0.001 (-0.066 to 0.064)	-8.626 (-17.166 to -0.086)	-0.068 (-0.136 to -0.001)
subthreshold	Concurrent	-0.521 (-8.923 to 7.881)	-0.002 (-0.034 to 0.030)	-4.706 (-21.551 to 12.139)	-0.009 (-0.043 to 0.024)
	Prospective	-0.826 (-3.926 to 2.273)	-0.007 (-0.036 to 0.021)	-5.097 (-11.212 to 1.018)	-0.024 (-0.053 to 0.005)
Bulimia nervosa	Baseline	1.504 (-3.983 to 6.990)	0.018 (-0.048 to 0.084)	-9.042 (-20.322 to 2.238)	-0.058 (-0.131 to 0.014)
subthreshold	Concurrent	2.533 (-11.500 to 16.565)	0.006 (-0.026 to 0.037)	16.479 (-11.671 to 44.629)	0.019 (-0.014 to 0.052)
	Prospective	-2.493 (-6.602 to 1.616)	-0.016 (-0.041 to 0.010)	-1.974 (-10.382 to 6.434)	-0.007 (-0.035 to 0.022)
Purging disorder	Baseline	-1.067 (-9.100 to 6.965)	-0.009 (-0.081 to 0.062)	5.977 (-12.643 to 24.597)	0.026 (-0.055 to 0.107)
	Concurrent	-4.088 (-8.380 to 0.203)	-0.024 (-0.049 to 0.001)	-0.865 (-8.386 to 6.657)	-0.003 (-0.029 to 0.023)
	Prospective	1.698 (-2.087 to 5.483)	0.011 (-0.014 to 0.036)	-2.732 (-9.778 to 4.315)	-0.010 (-0.035 to 0.015)
Night eating	Baseline	0.797 (-5.873 to 7.468)	0.009 (-0.066 to 0.084)	7.897 (-6.908 to 22.703)	0.043 (-0.038 to 0.123)

© 2022 Hilbert A et al. *JAMA Network Open.*

		Percent total body weight loss[a]		Health-related quality of life[a]	
		**Estimate (95% CI)**	**Std. estimate (95% CI)**	**Estimate (95% CI)**	**Std. estimate (95% CI)[a]**
syndrome	Concurrent	1.083 (-1.571 to 3.737)	0.011 (-0.016 to 0.038)	-3.220 (-8.265 to 1.825)	-0.018 (-0.045 to 0.010)
	Prospective	-0.898 (-3.300 to 1.503)	-0.009 (-0.035 to 0.016)	-4.086 (-9.181 to 1.009)	-0.020 (-0.046 to 0.005)
**Eating disorders based on loss of control eating[c]**					
Binge-eating disorder	Baseline	0.177 (-2.410 to 2.763)	0.004 (-0.059 to 0.068)	-7.821 (-13.331 to -2.311)	-0.101 (-0.172 to -0.030)
	Concurrent	-2.600 (-4.574 to -0.626)	-0.032 (-0.057 to -0.008)	-2.823 (-6.831 to 1.186)	-0.019 (-0.046 to 0.008)
	Prospective	-1.888 (-3.401 to -0.374)	-0.033 (-0.059 to -0.007)	-1.635 (-4.748 to 1.478)	-0.014 (-0.042 to 0.013)
Bulimia nervosa	Baseline	3.016 (-3.138 to 9.170)	0.036 (-0.037 to 0.109)	-13.243 (-25.914 to -0.571)	-0.084 (-0.164 to -0.004)
	Concurrent	-4.743 (-9.376 to -0.110)	-0.026 (-0.051 to -0.001)	3.181 (-6.147 to 12.510)	0.009 (-0.018 to 0.036)
	Prospective	-5.098 (-8.454 to -1.742)	-0.037 (-0.062 to -0.013)	2.678 (-3.805 to 9.160)	0.010 (-0.015 to 0.036)
Binge-eating disorder	Baseline	0.252 (-2.607 to 3.111)	0.006 (-0.062 to 0.074)	-5.498 (-11.384 to 0.387)	-0.066 (-0.137 to 0.005)
subthreshold	Concurrent	0.317 (-1.403 to 2.037)	0.005 (-0.021 to 0.030)	-3.093 (-6.477 to 0.291)	-0.024 (-0.051 to 0.002)
	Prospective	-0.986 (-2.468 to 0.496)	-0.017 (-0.044 to 0.009)	-4.466 (-7.398 to -1.533)	-0.041 (-0.069 to -0.014)
Bulimia nervosa	Baseline	1.935 (-9.915 to 13.785)	0.012 (-0.062 to 0.086)	-25.360 (-48.000 to -2.720)	-0.081 (-0.153 to -0.009)
subthreshold[b]	Prospective	-3.347 (-12.827 to 6.134)	-0.013 (-0.049 to 0.023)	15.810 (-3.554 to 35.173)	0.025 (-0.006 to 0.056)

Abbreviations: DSM-5, Diagnostic and Statistical Manual of Mental Disorders 5th Edition.[9]

[a]Unstandardized and standardized estimates with 95% confidence intervals from multivariable longitudinal linear mixed regression models without adjustment for age, sex, baseline weight, surgical procedure, and reoperations on nonnormative eating behaviors or eating disorder diagnoses in their concurrent and prospective associations with percent total body weight loss and health-related quality of life, assessed with the Impact of Weight on Quality of Life-Lite total score (0=*worst* to 100=*best*).[11,12]

[b]Not detected at across follow-up, therefore concurrent models were not computed, and baseline effects were displayed from prospective models.

[c]In an exploratory analysis, eating disorders were diagnosed based on loss of control eating instead of objective binge eating as required by the DSM-5. For comparability, the explorative models additionally included all other DSM-5 eating disorder diagnoses.

© 2022 Hilbert A et al. *JAMA Network Open.*

eTable 5. Sensitivity Analysis for Length of Follow-up: Percent Total Body Weight Loss and Health-related Quality of Life with Nonnormative Eating Behaviors or Eating Disorders According to DSM-5 Over 6 Years Following Obesity Surgery for Patients with Follow-ups of 2 Years or More

		Percent total body weight loss[a]		Health-related quality of life[a]	
		Estimate (95% CI)	Std. estimate (95% CI)	Estimate (95% CI)	Std. estimate (95% CI)
**Nonnormative eating behaviors**					
Loss of control eating	Baseline	0.011 (-0.063 to 0.085)	0.013 (-0.072 to 0.097)	-0.028 (-0.277 to 0.222)	-0.009 (-0.091 to 0.073)
	Concurrent	-0.078 (-0.133 to -0.023)	-0.039 (-0.067 to -0.012)	-0.123 (-0.217 to -0.029)	-0.044 (-0.078 to -0.010)
	Prospective	-0.004 (-0.041 to 0.033)	-0.004 (-0.033 to 0.026)	-0.138 (-0.219 to -0.058)	-0.052 (-0.083 to -0.022)
Objective binge eating	Baseline	-0.007 (-0.084 to 0.069)	-0.008 (-0.091 to 0.075)	0.084 (-0.269 to 0.438)	0.019 (-0.061 to 0.099)
	Concurrent	-0.061 (-0.136 to 0.013)	-0.022 (-0.049 to 0.005)	-0.156 (-0.297 to -0.015)	-0.031 (-0.059 to -0.003)
	Prospective	0.011 (-0.030 to 0.053)	0.008 (-0.022 to 0.038)	-0.162 (-0.287 to -0.036)	-0.035 (-0.062 to -0.008)
Subjective binge eating	Baseline	0.249 (-0.009 to 0.508)	0.083 (-0.003 to 0.170)	-0.146 (-0.525 to 0.234)	-0.030 (-0.109 to 0.049)
	Concurrent	-0.106 (-0.187 to -0.025)	-0.038 (-0.066 to -0.009)	-0.099 (-0.221 to 0.023)	-0.030 (-0.066 to 0.007)
	Prospective	-0.059 (-0.132 to 0.015)	-0.022 (-0.049 to 0.005)	-0.124 (-0.226 to -0.022)	-0.038 (-0.070 to -0.007)
Night eating	Baseline	0.004 (-0.095 to 0.102)	0.003 (-0.088 to 0.095)	-0.076 (-0.249 to 0.097)	-0.034 (-0.110 to 0.043)
	Concurrent	0.035 (-0.016 to 0.086)	0.020 (-0.009 to 0.050)	-0.036 (-0.121 to 0.050)	-0.014 (-0.049 to 0.020)
	Prospective	-0.018 (-0.065 to 0.029)	-0.012 (-0.043 to 0.019)	-0.055 (-0.133 to 0.023)	-0.022 (-0.054 to 0.009)
Compensatory behaviors	Baseline	-0.202 (-0.441 to 0.038)	-0.076 (-0.166 to 0.014)	0.049 (-0.292 to 0.389)	0.010 (-0.060 to 0.081)
	Concurrent	0.015 (-0.106 to 0.137)	0.004 (-0.025 to 0.032)	-0.058 (-0.278 to 0.162)	-0.008 (-0.037 to 0.022)
	Prospective	-0.047 (-0.151 to 0.057)	-0.012 (-0.040 to 0.015)	0.038 (-0.156 to 0.232)	0.006 (-0.023 to 0.034)
**DSM-5 Eating disorders**					
Binge-eating disorder	Baseline	0.963 (-3.436 to 5.361)	0.019 (-0.066 to 0.104)	-0.937 (-8.509 to 6.636)	-0.009 (-0.086 to 0.067)
	Concurrent	-0.335 (-5.182 to 4.512)	-0.002 (-0.029 to 0.025)	-2.986 (-11.949 to 5.976)	-0.009 (-0.036 to 0.018)
	Prospective	-1.067 (-3.634 to 1.500)	-0.011 (-0.039 to 0.016)	-1.756 (-7.079 to 3.567)	-0.009 (-0.037 to 0.018)
Bulimia nervosa[b]	Baseline	3.246 (-4.840 to 11.332)	0.034 (-0.051 to 0.120)	-7.465 (-22.658 to 7.728)	-0.036 (-0.110 to 0.037)
	Prospective	-0.643 (-6.024 to 4.739)	-0.003 (-0.029 to 0.023)	7.025 (-5.004 to 19.053)	0.016 (-0.011 to 0.043)
Binge-eating disorder subthreshold	Baseline	0.897 (-4.227 to 6.020)	0.015 (-0.069 to 0.098)	1.466 (-7.306 to 10.237)	0.012 (-0.058 to 0.081)
	Concurrent	-0.141 (-8.693 to 8.410)	-0.001 (-0.037 to 0.036)	-6.065 (-22.170 to 10.041)	-0.014 (-0.052 to 0.023)
	Prospective	-1.452 (-4.904 to 2.001)	-0.012 (-0.040 to 0.016)	-8.717 (-15.751 to -1.683)	-0.036 (-0.065 to -0.007)
Bulimia nervosa subthreshold	Baseline	2.183 (-12.090 to 16.456)	0.005 (-0.030 to 0.041)	4.644 (-6.283 to 15.571)	0.032 (-0.043 to 0.106)
	Concurrent	2.913 (-3.921 to.9.747)	0.036 (-0.048 to 0.119)	19.322 (-7.620 to 46.265)	0.026 (-0.010 to 0.062)
	Prospective	-3.520 (-8.010 to 0.970)	-0.021 (-0.048 to 0.006)	-5.812 (-14.558 to 2.935)	-0.018 (-0.046 to 0.009)
Purging disorder	Baseline	-1.727 (-11.054 to 7.600)	-0.016 (-0.105 to 0.072)	5.462 (-9.170 to 20.093)	0.027 (-0.046 to 0.101)
	Concurrent	-4.538 (-8.908 to -0.169)	-0.030 (-0.058 to -0.001)	-0.293 (-7.842 to 7.256)	-0.001 (-0.030 to 0.028)
	Prospective	2.442 (-1.663 to 6.548)	0.016 (-0.011 to 0.042)	-0.118 (-7.236 to 7.000)	0.000 (-0.028 to 0.027)

© 2022 Hilbert A et al. *JAMA Network Open.*

		Percent total body weight loss[a]		Health-related quality of life[a]	
		**Estimate (95% CI)**	**Std. estimate (95% CI)**	**Estimate (95% CI)**	**Std. estimate (95% CI)**
Night eating	Baseline	0.133 (-6.843 to 7.110)	0.002 (-0.085 to 0.089)	3.607 (-8.176 to 15.389)	0.023 (-0.051 to 0.096)
syndrome	Concurrent	2.662 (-0.309 to 5.632)	0.026 (-0.003 to 0.055)	-3.713 (-8.532 to 1.106)	-0.023 (-0.053 to 0.007)
	Prospective	0.541 (-2.099 to 3.181)	0.006 (-0.022 to 0.034)	-4.295 (-9.185 to 0.595)	-0.025 (-0.053 to 0.003)
**Eating disorders based on loss of control eating[c]**					
Binge-eating disorder	Baseline	-0.133 (-3.528 to 3.263)	-0.003 (-0.085 to 0.078)	-4.810 (-10.301 to 0.681)	-0.064 (-0.136 to 0.009)
	Concurrent	-1.886 (-3.981 to 0.209)	-0.024 (-0.052 to 0.003)	-2.104 (-6.029 to 1.821)	-0.016 (-0.045 to 0.014)
	Prospective	-1.868 (-3.584 to -0.153)	-0.030 (-0.058 to -0.002)	-1.959 (-5.221 to 1.303)	-0.018 (-0.047 to 0.012)
Bulimia nervosa	Baseline	2.737 (-4.192 to 9.665)	0.035 (-0.053 to 0.123)	1.770 (-9.038 to 12.578)	0.012 (-0.064 to 0.089)
	Concurrent	-4.705 (-9.795 to 0.385)	-0.026 (-0.054 to 0.002)	3.166 (-5.782 to 12.114)	0.010 (-0.019 to 0.040)
	Prospective	-5.267 (-8.813 to -1.721)	-0.041 (-0.068 to -0.013)	2.319 (-4.147 to 8.785)	0.010 (-0.018 to 0.038)
Binge-eating disorder	Baseline	-0.075 (-3.711 to 3.561)	-0.002 (-0.089 to 0.086)	-0.976 (-7.003 to 5.050)	-0.012 (-0.085 to 0.061)
subthreshold	Concurrent	0.542 (-1.322 to 2.406)	0.008 (-0.020 to 0.036)	-1.781 (-5.339 to 1.777)	-0.014 (-0.043 to 0.014)
	Prospective	-1.115 (-2.743 to 0.513)	-0.019 (-0.048 to 0.009)	-3.264 (-6.453 to -0.075)	-0.029 (-0.058 to -0.001)
Bulimia nervosa	Baseline	1.786 (-14.479 to 18.052)	0.011 (-0.087 to 0.108)	20.713 (-5.542 to 46.967)	0.066 (-0.018 to 0.149)
subthreshold[b]	Prospective	-2.470 (-12.966 to 8.026)	-0.006 (-0.032 to 0.020)	11.883 (-8.502 to 32.267)	0.015 (-0.011 to 0.042)

Abbreviations: DSM-5, Diagnostic and Statistical Manual of Mental Disorders 5th Edition.[9]
[a]Unstandardized and standardized estimates with 95% confidence intervals from multivariable longitudinal linear mixed regression models of nonnormative eating behaviors or eating disorder diagnoses in their concurrent and prospective associations with percent total body weight loss and health-related quality of life, assessed with the Impact of Weight on Quality of Life-Lite total score (0=*worst* to 100=*best*).[11,12]
[b]Not detected at across follow-up, therefore concurrent models were not computed, and baseline effects were displayed from prospective models.
[c]In an exploratory analysis, eating disorders were diagnosed based on loss of control eating instead of objective binge eating as required by the DSM-5. For comparability, the explorative models additionally included all other DSM-5 eating disorder diagnoses.

© 2022 Hilbert A et al. *JAMA Network Open.*
